# Effect of three oral pathogens on the TMA-TMAO metabolic pathway

**DOI:** 10.3389/fcimb.2024.1413787

**Published:** 2024-05-21

**Authors:** Xixuan Wang, Liyuan Chen, Ye Teng, Weige Xie, Lingyan Huang, Juan Wu, Hongwei Wang, Sijing Xie

**Affiliations:** ^1^ Nanjing Stomatological Hospital, Affiliated Hospital of Medical School, Nanjing University, Nanjing, China; ^2^ Nantong Stomatological Hospital, Affiliated Nantong Stomatological Hospital of Nantong University, Nantong, China; ^3^ Center for Translational Medicine and Jiangsu Key Laboratory of Molecular Medicine, Medical School of Nanjing University, Nanjing, China

**Keywords:** Porphyromonas gingivalis, Fusobacterium nucleatum, Streptococcus mutans, trimethylamine N-oxide, flavin-containing monooxygenase

## Abstract

**Background:**

Trimethylamine-N-oxide (TMAO) is produced by hepatic flavin-containing monooxygenase 3 (FMO3) from trimethylamine (TMA). High TMAO level is a biomarker of cardiovascular diseases and metabolic disorders, and it also affects periodontitis through interactions with the gastrointestinal microbiome. While recent findings indicate that periodontitis may alter systemic TMAO levels, the specific mechanisms linking these changes and particular oral pathogens require further clarification.

**Methods:**

In this study, we established a C57BL/6J male mouse model by orally administering *Porphyromonas gingivalis* (*P. gingivalis*, *Pg*), *Fusobacterium nucleatum* (*F. nucleatum*, *Fn*), *Streptococcus mutans* (*S. mutans*, *Sm*) and PBS was used as a control. We conducted LC-MS/MS analysis to quantify the concentrations of TMAO and its precursors in the plasma and cecal contents of mice. The diversity and composition of the gut microbiome were analyzed using 16S rRNA sequencing. TMAO-related lipid metabolism and enzymes in the intestines and liver were assessed by qPCR and ELISA methods. We further explored the effect of *Pg* on FMO3 expression and lipid molecules in HepG2 cells by stimulating the cells with *Pg*-LPS *in vitro*.

**Results:**

The three oral pathogenic bacteria were orally administered to the mice for 5 weeks. The *Pg* group showed a marked increase in plasma TMAO, betaine, and creatinine levels, whereas no significant differences were observed in the gut TMAO level among the four groups. Further analysis showed similar diversity and composition in the gut microbiomes of both the *Pg* and *Fn* groups, which were different from the *Sm* and control groups. The profiles of TMA-TMAO pathway-related genera and gut enzymes were not significantly different among all groups. The *Pg* group showed significantly higher liver FMO3 levels and elevated lipid factors (IL-6, TG, TC, and NEFA) in contrast to the other groups. *In vitro* experiments confirmed that stimulation of HepG2 cells with *Pg*-LPS upregulated the expression of FMO3 and increased the lipid factors TC, TG, and IL-6.

**Conclusion:**

This study conclusively demonstrates that *Pg*, compared to *Fn* and *Sm*, plays a critical role in elevating plasma TMAO levels and significantly influences the TMA-TMAO pathway, primarily by modulating the expression of hepatic FMO3 and directly impacting hepatic lipid metabolism.

## 1 Introduction

The oral cavity serves as a significant microbial reservoir within the human body. Studies have established links between oral diseases, especially periodontitis, and various systemic diseases, including cardiovascular diseases (CVD) (Genco and Sanz, 2020), inflammatory bowel disease (IBD) (Kul et al., 2023), non-alcoholic fatty liver disease (NAFLD) (Kuraji et al., 2021), cancer, Alzheimer’s disease (AD) (Lu et al., 2022), and rheumatoid arthritis (RA) (Chan et al., 2019) etc.

Dental plaque is the primary cause of oral diseases (Simon-Soro et al., 2022). Species such as *Streptococcus mutans* (*Sm*) are the initial inhabitants of dental plaque, adhering through mechanisms mediated by RadD and other mechanisms (Kolenbrander et al., 2010; Baty et al., 2022). RadD is a protein encoded by the FNP_1046 gene in *Fusobacterium nucleatum* (*Fn*), it promotes adhesion by recognizing and binding to partner adhesins on other species, such as SpaP on *Sm*, as demonstrated in heterologous expression systems (Guo et al., 2017). Subsequently, *Fn* plays a central bridging role in the formation of oral biofilms, uniquely positioned to interact with both early and late colonizers such as *Sm* and *Porphyromonas gingivalis* (*Pg*) in the oral cavity (Kaplan et al., 2009; Valadbeigi et al., 2023). Anaerobic bacteria like *Pg* are subsequently incorporated into the biofilm through adhesins such as Fap2, thereby exacerbating oral diseases (Mark Welch et al., 2016; Kim and Davey, 2020; Verma et al., 2023). Several studies have explored these oral pathogenic bacteria due to their potential role in linking oral and systemic diseases. These pathogens can cause systemic diseases through hematogenous (Lei et al., 2023) or intestinal pathways (Buetas et al., 2024; Wang et al., 2022).

Research has demonstrated that the progression of systemic disease is significantly influenced by gut microbiota and their metabolites through the intestinal pathway (Blasco-Baque et al., 2017; Lang and Schnabl, 2020). Trimethylamine-N-oxide (TMAO) has been recognized for its potential toxicity (Spence, 2023). Dietary sources such as carnitine, choline, creatinine, and L-carnitine are metabolized by intestinal bacteria into trimethylamine (TMA), which is then absorbed by intestinal epithelial cells. From there, TMA enters the bloodstream, is transported to the liver via the portal circulation, and is oxidized by host enzyme called hepatic flavin-containing monooxygenase 3 (FMO3) into TMAO (Shih et al., 2015). This pathway *in vitro* involves members of the genera *Anaerococcus*, *Clostridium*, *Escherichia*, *Proteus*, *Providencia*, and *Edwardsiella* (Romano et al., 2015), which commonly harbor the CutC and CutD genes (Canyelles et al., 2023). These genes encode bacterial enzymes crucial for the cleavage of choline to TMA. And its effect on TMA production can differ significantly across individuals in clinical research (Martínez-del Campo et al., 2015), often influenced by dietary patterns. Multivariate analyses have identified several bacterial species whose abundance correlates significantly with plasma TMAO levels but not necessarily with TMA levels in US men, indicating distinct microbial contributions under different dietary conditions (Li et al., 2022).

Studies now show a marked correlation between TMAO levels and CVD (He et al., 2023; Spence, 2023). In murine models, TMAO has been shown to promote atherosclerosis (AS) through mechanisms such as enhancing the formation of cholesterol-loaded macrophage foam cells, which are central to the development of atherosclerotic plaques (Wang et al., 2011). Furthermore, a significant study involving over 4,000 individuals undergoing coronary angiography revealed that those with TMAO levels in the highest quartile faced a 2.5 times greater risk of experiencing myocardial infarction, stroke, or vascular death within a span of three years (Tang et al., 2013). Apart from these, previous findings also indicate that TMAO is also a prognostic marker for other metabolic diseases such as myocardial infarction (MI), obesity, diabetes, NAFLD (Chen et al., 2016; Flores-Guerrero et al., 2021).

Recently, studies highlighted a potential association between oral diseases and TMAO. *Streptococcus sanguinis* (*Ss*) is a producer of TMAO in the oral cavity, potentially amplifying the gut microbiome’s role in exacerbating AS by enhancing TMA production (Chao and Zeisel, 1990). Significantly, elevated concentrations of TMAO have been observed in individuals with periodontitis (stages III-IV), correlating with endothelial dysfunction (Zhou et al., 2022). Plasma TMAO levels were higher in ApoE^−/−^ mice infected with *Pg* via the dental pulp (Gan et al., 2023), thereby implying that periodontitis may lead to elevated plasma TMAO levels. In addition, TMAO may have an effect on the progression of periodontitis via the two-way interaction between periodontitis and gastrointestinal microbiomes (Wang Q. et al., 2022). Preliminary research revealed changes in gut microbiome and liver status in ApoE^−/−^ mice within an experimental periodontitis cohort, accompanied by a rise in plasma TMAO levels (Xiao et al., 2022). Additionally, clinical studies conducted by our team have also found that patients with both AS and periodontitis exhibit higher peripheral blood levels of TMAO compared to patients without periodontitis (Wang et al., 2023). However, the relationship among these changes and specific oral pathogens or the local immune response has not been elucidated.

In this study, we selected *Pg* and *Fn* for their well-established roles in periodontal disease and systemic health, with *Sm* serving as a comparative non-periodontal pathogen control. Building upon preliminary research linking TMAO levels to periodontitis (Xiao et al., 2022), our goal was to evaluate the effect of specific periodontal pathogens on TMAO production in mice fed a standard diet, as opposed to host inflammatory responses, and to investigate the underlying mechanisms influencing the TMA-TMAO metabolic pathway.

## 2 Materials and methods

### 2.1 Mice

Twenty male SPF C57BL/6J mice, aged 8 weeks, were utilized for the study. After a 2-week acclimatization period, the mice were allocated into four groups in a random way. Each group received oral administration of a pathogenic bacterial suspension (*Pg*, *Fn*, or *Sm*) or PBS separately twice weekly for five weeks, and the plasma, cecal contents, small intestine, large intestine, and liver of the mice were collected. Experimental conditions were regulated to ensure a stable environment with a temperature of 25 ± 2°C, relative humidity at 55% ± 10%, and a consistent 12-hour light/12-hour dark cycle. The mice were sourced from the Model Animal Research Institute of Nanjing University (China, Nanjing), and protocols were conducted with the approval of the Nanjing University Animal Experiment Ethics Committee (Approval No. IACUC-D2102033).

### 2.2 Bacterial cultures

*Porphyromonas gingivalis ATCC33277*, *Fusobacterium nucleatum ATCC 25586*, and *Streptococcus mutans ATCC 25175* were obtained from the Central Laboratory of Nanjing University Affiliated Stomatological Hospital. Following thawing, the bacteria were separately cultured in brain-heart infusion broth (BHI) (HopeBio, China) supplemented with vitamin K (1 μg/mL, Solarbio, China), hemin (5 μg/mL, Solarbio, China), and yeast extraction (OXOID, UK) at 37°C under anaerobic conditions for 48 hours. Optical density (OD) measurements were conducted at a wavelength of 600 nm, yielding an OD value of 0.9. Each pathogen (1× 109 CFUs/mL) was suspended in 200 µL of phosphate-buffered saline (PBS) containing 2% carboxymethyl cellulose (Sigma, USA), and the suspension was oral administered to the mice.

### 2.3 LC-MS/MS-based TMAO metabolome quantification

Frozen cecal content samples were mixed with an internal standard and methanol-water, followed by homogenization and extraction using ultrasonication. The supernatant was dried after centrifugation, reconstituted, and prepared for metabolite analysis using LC-MS/MS. The resulting supernatant (150 µL) was transferred to an LC-MS vial containing a conical insert for quantitative analysis of metabolites including TMAO, TMA, betaine, choline, creatinine, and L- carnitine.

### 2.4 Histological analysis of liver samples

Liver samples were stained by covering the sections with staining solution for 10–15 minutes. Subsequently, any excess dye was eliminated using 60% isopropanol, followed by a thorough rinsing with distilled water. Finally, the coverslips were placed on the slides, and an image analysis was performed for evaluation.

### 2.5 Intestinal and hepatic gene expression analysis

After the hepatic and intestinal tissues of mice were collected, total RNA was isolated utilizing the FastPure^®^ Tissue Total RNA Isolation Kit V2 (Vazyme, China). Subsequent reverse transcription PCR was executed with HiScript^®^ III RT SuperMix for qPCR (+gDNA wiper) (Vazyme, China). Primers for the qPCR were acquired from GenScript, China. The qPCR itself was carried out on the LightCyclerH 96 system (Roche). The relative expression levels of IL-6, IL-1β, TNF-α, and the FMO gene family were quantified by normalizing to ACTB mRNA expression, employing the 2^−ΔΔCT^ method for data analysis.

### 2.6 Detection of oral pathogenic bacteria in liver and intestinal samples

Bacterial DNA was isolated from hepatic and cecal samples utilizing the FastPure^®^ Microbiome DNA Isolation Kit (Vazyme, Nanjing, China). The quantification of real-time PCR was conducted with ChamQ Universal SYBR qPCR Master Mix (Vazyme, China), then the LightCyclerH 96 system (Roche) was used. Amplification of the 16S rRNA gene segments specific to universal bacteria, as well as *Pg*, *Fn*, and *Sm*, was achieved through the employment of targeted primers.

### 2.7 16S rRNA sequencing and gut microbiota analysis

DNA extraction from mouse cecal content samples was performed utilizing the TIANamp Stool DNA Kit (Tiangen, China), followed by analysis of the composition of gut microbiota. Universal primers (Forward: F: TACGGRAGGCAGCAG. Reverse: R: AGGGTATCTAATCCT) were used for PCR amplification of the extracted DNA. Subsequently, a bioinformatics analysis of the amplified products was conducted on the OECloud online analysis platform (https://cloud.oebiotech.cn) to explore the bacterial community structure within the cecal contents.

### 2.8 Biochemical analysis of liver function parameters

Commercial assay kits (Nanjing Jiancheng Bioengineering Institute, China) were used to evaluate the concentrations of alanine transaminase (ALT), aspartate transaminase (AST), total cholesterol (TC), triglycerides (TG), and non-esterified fatty acids (NEFA) in homogenates of mouse liver, in accordance with the prescribed procedural guidelines.

### 2.9 Choline TMA-lyase (CutC, CutD) immunoassay

The contents of TMA-lyase CutC and CutD in mouse cecal contents were determined using commercial ELISA kits (Sihan Biotech, China). The concentrations of CutC and CutD were determined using the standard curve.

### 2.10 Cell culture

HepG2 cells were cultured in DMEM medium supplemented with high glucose, 10% fetal bovine serum, and antibiotics (consisting of 100 U/mL penicillin and 100 µg/mL streptomycin). Cells were cultured at 37°C with 5% CO2 in a in a humidified atmosphere. Then, the cells were divided into three groups for treatment: a control group, a group exposed to 10 µg/mL *Pg*-LPS, and a third group exposed to 10 µg/mL *E.coli*-LPS, each for a duration of 24 hours.

### 2.11 Data analysis

Data from our results were noted as the mean ± SEM and subjected to one-way ANOVA analysis using GraphPad Prism 10 (GraphPad Software, San Diego, California, USA). Significance were established at *p <0.05, **p <0.01, ***p < 0.001, and ****p < 0.001 to evaluate intergroup differences.

## 3 Results

### 3.1 *Pg* induced elevated levels of the toxic metabolite TMAO in the peripheral blood of mice

**Figure 1:**
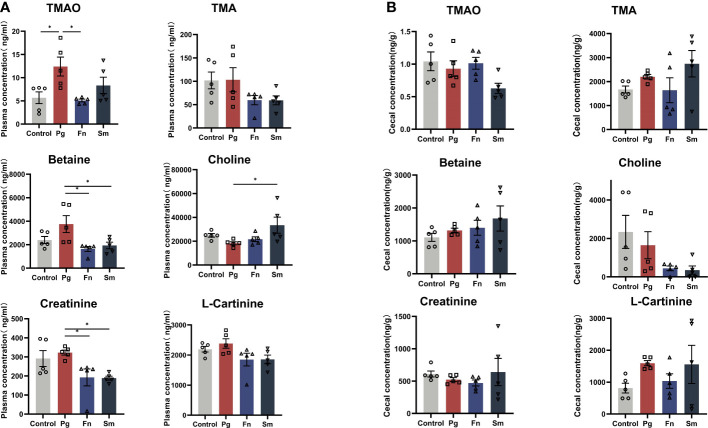
Effect of three oral pathogens on TMA metabolism in C57BL/6J mice. **(A)** Plasma concentrations of TMAO, TMA, betaine, choline, creatinine, and L- carnitine measured using liquid chromatography-mass spectrometry/mass spectrometry (LC-MS/MS). **(B)** Concentrations of TMAO, TMA, betaine, choline, creatinine, and L- carnitine in cecal contents determined using LC-MS/MS. Significance levels are indicated as follows: *p<0.05.

*Pg*, *Fn*, *Sm*, and PBS were orally administered to 8-week-old C57BL/6J mice twice per week, with each treatment administered separately. After 5 weeks, plasma samples and cecal contents samples were analyzed to determine the concentration of TMAO and other metabolites. The TMA precursors such as betaine, choline, creatinine, and L-Carnitine are metabolized by gut microbiota to form TMA. These molecules were measured to understand the full scope of the metabolic effect that oral pathogens may have on the host. The analysis revealed a notable increase in the plasma TMAO concentration in the *Pg* group compared with the control group (**Figure 1A**), whereas no significant difference in TMA level was observed across the four groups. A decrease in the levels of the precursor substances, betaine and creatinine, was observed in the plasma of the *Fn* and *Sm* groups compared to the *Pg* group. Elevated levels of choline in the *Pg* group were observed as statistically significant when compared to the *Sm* group, while the levels of L-carnitine remained consistent across all groups. However, there were no significant differences in the concentrations of TMAO and TMA precursor substances in the cecal contents among the four groups (**Figure 1B**).

### 3.2 *Pg* and *Fn* modulates the diversity and structure of gut microbiota

**Figure 2:**
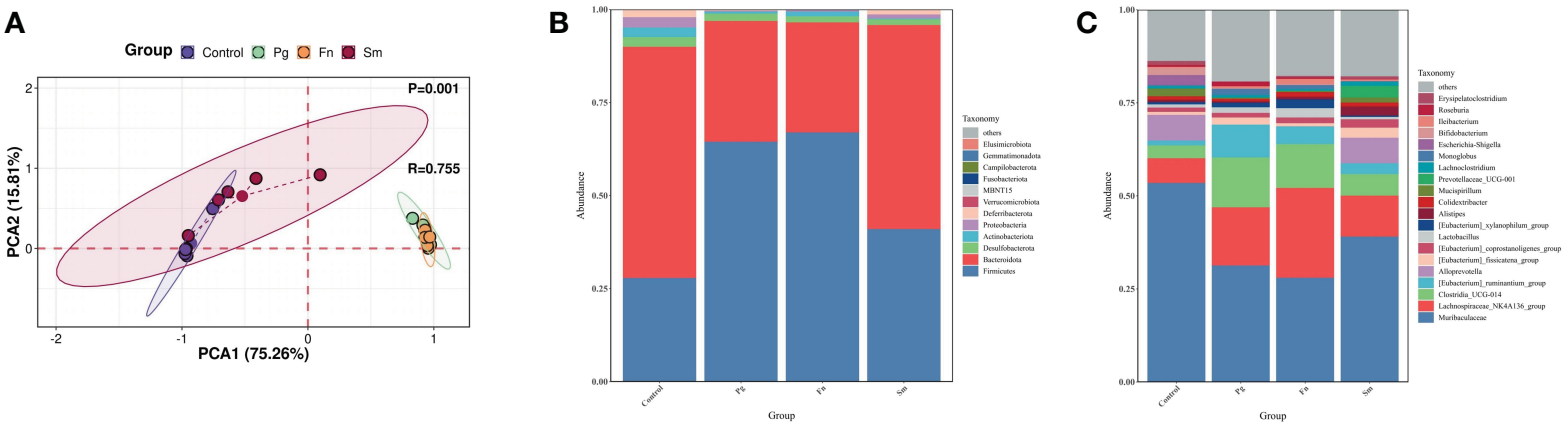
Effect of three oral pathogens on the diversity and composition of gut microbiota, as analyzed through 16S rRNA bioinformatics. **(A)** Beta-diversity analysis of intestinal microbiota across different treatment groups. **(B)** Relative abundance of microbiota at the phylum level. **(C)** Relative abundance at the genus level in gut microbiota.

TMAO originates from TMA and its precursors in the gut (Canyelles et al., 2023). Therefore, a comprehensive analysis was conducted to assess the diversity and composition of the gut microbiome. PCoA analysis revealed a significant overlap between the *Pg* and *Fn* groups, as well as between the *Sm* and control groups (*P*=0.001) (**Figure 2A**). The Shannon and Simpson indices of intestinal microbiota were higher in the *Sm* group compared to the control group. However, no significant differences were observed in these indices among the *Pg*, *Fn*, and control groups (**Supplementary Figure 1**).

At the phylum level, our analysis of gut microbiome composition indicated that the *Bacteroidota*/*Firmicutes* ratio was lower in the *Pg* and *Fn* groups compared to the *Sm* and control groups (**Figure 2B**). The *Pg* and *Fn* groups also exhibited reduced proportions of other phyla, such as *Deferribacterota* and *Proteobacteria*. The relative abundance of microbiota in the *Sm* group was similar to that in the control group. At the genus level, a decrease in the relative abundance of genera, including *Muribaculaceae* and *Alloprevotella* etc, was observed in the *Pg* and *Fn* group. In contrast, genera such as *Lachnospiraceae_NK4A136_group* and *Clostridia UCG-014* showed an increase which was also observed in the *Pg* and *Fn* group. As mentioned above, the relative abundance of key microbial taxa, particularly the genera *Lachnospiraceae_NK4A136_group* and *Clostridia UCG-014*, showed a slight but discernible alteration in the *Sm* and control groups, reflecting subtle shifts in gut microbial diversity and potential metabolic effect (**Figure 2C**).

The distinct bacterial genera among the groups were identified, and the potential effects of gut functional changes in these genera were evaluated using LEfSe analysis (**Supplementary Figure 2**) and Random Forest analysis (**Supplementary Figure 3**). *Parvibacter*, *Alistipes*, and *Rikenella* were identified as the most distinct genera across all four groups.

### 3.3 The levels of TMA-related bacterial genera and related enzymes were similar in the *Pg* and *Fn* groups compared with the control group

**Figure 3:**
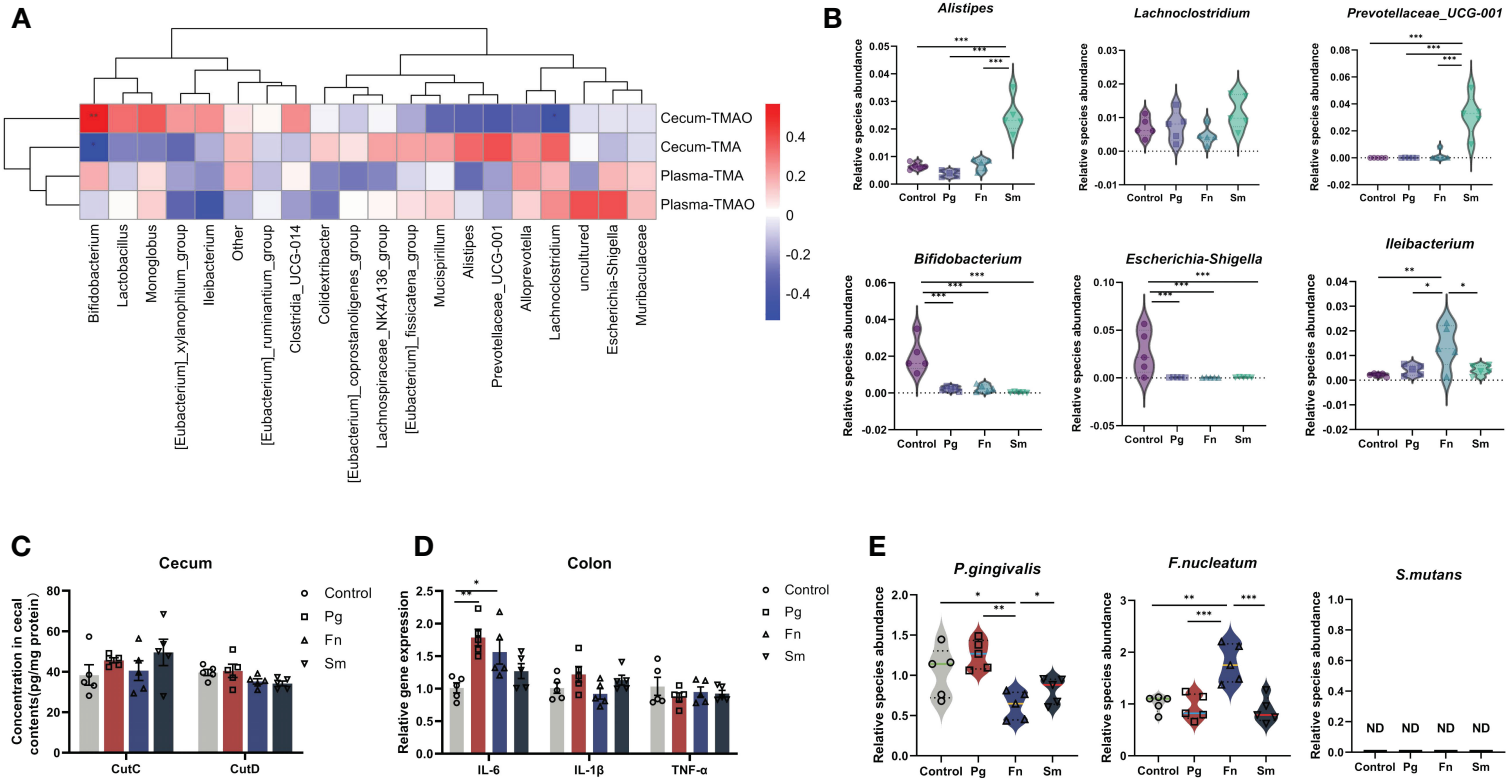
Effect of three oral pathogens on TMAO-related gut microbiota. **(A)** Correlations between intestinal bacterial genera and levels of TMAO and TMA, analyzed using Spearman correlation analysis (0–0.3 indicate weak, 0.3–0.6 indicates moderate, and 0.6–1.0 indicates strong correlation). **(B)** Relative abundance of gut microbiota associated with TMAO and TMA. **(C)** Concentration of choline TMA-lyases CutC and CutD in cecal content, measured via enzyme-linked immunosorbent assay (ELISA). **(D)** Relative gene expression levels of intestinal inflammatory factors, quantified through quantitative polymerase chain reaction (qPCR). **(E)** Relative abundance of oral pathogens in the intestine, determined by qPCR. Significance levels indicated: *p<0.05, **p<0.01, ***p<0.001.

We performed a Spearman correlation analysis of between the top 20 bacterial genera at the genus level and TMAO metabolic production to identify the bacterial genera involved in TMAO-related metabolic pathways (**Figure 3A**). In our study, the selection of the top 20 genera was based on their relative abundance across all groups—both control and infected—from a 16S rRNA dataset.

The results indicated a positive modest correlation between *Escherichia-Shigella* and plasma TMAO levels, whereas a modest negative correlation was observed between *Ileibacterium* and plasma TMAO. Similarly, we observed a weak positive correlation between *Escherichia-Shigella* and plasma TMA, and a weak negative correlation with *Ileibacterium*. Additionally, cecum TMA levels demonstrated a positive moderate correlation with the relative abundance of *Prevotellaceae_UCG-001*, *Alistipes*, and *Lachnoclostridium*. Conversely, a negative moderate correlation was observed between cecum TMA levels and the relative abundance of *Bifidobacterium*. Furthermore, cecum TMAO production exhibited a negative correlation with TMA levels.

Then, six bacterial genera were identified based on their moderate correlation with TMAO metabolism in **Figure 3A**, as indicated by Spearman correlation coefficients ranging from 0.3 to 0.6. A significant increase in the relative abundance of *Prevotellaceae_UCG-001* and *Alistipes* was observed in the *Sm* group compared to the control group. Conversely, a significant reduction in the abundance of *Bifidobacterium* was observed across all three microbial groups (*Pg*, *Fn*, and *Sm*) in contrast with control group. Moreover, a significant decrease in the abundance of *Escherichia-Shigella* was observed in all three bacterial groups, whereas *Ileibacterium* exhibited an increase in abundance only in the *Fn* group (**Figure 3B**).

CutC and CutD are TMA lyases affected by gut microbiota and indicate the capacity of the intestinal microbiota to produce TMA (Canyelles et al., 2023). These parameters were assessed in the cecal contents of the four groups. The results revealed no significant difference in CutC and CutD concentrations among the four groups. (**Figure 3C**).

Then, we selected IL-6, IL-1β, and TNF-α for measurement due to their pivotal roles in mediating inflammatory responses. IL-6 expression levels were significantly higher in the *Pg* and *Fn groups* compared to the *Sm* and control groups, indicating increased inflammation (**Figure 3D**). Additionally, in this study, ZO-1 and occludin were chosen due to their essential roles in maintaining tight junction integrity and regulating epithelial barrier permeability. These proteins are critical for the prevention of unwanted substances from passing through the gut barrier. The *Pg* group exhibited a reduction in the content of the occludin barrier protein (**Supplementary Figure 4**), while the *Fn* and *Sm* groups showed slightly difference compared to the control group. Although changes in ZO-1 expression were also monitored, the alterations were not statistically significant across the different treatment groups (*p* >0.05). H&E staining further demonstrated that the *Pg* group had the most severe intestinal damage among all the groups (**Supplementary Figure 5**).

After oral administration, the abundance of *Fn* in the intestine increased significantly, when there were no significant changes in the relative abundance of *Pg* in the intestine. Only *Sm* was not detected (**Figure 3E**).

### 3.4 *Pg* promotes upregulation of FMO3 expression in the liver of mice and fatty degeneration of the liver

**Figure 4:**
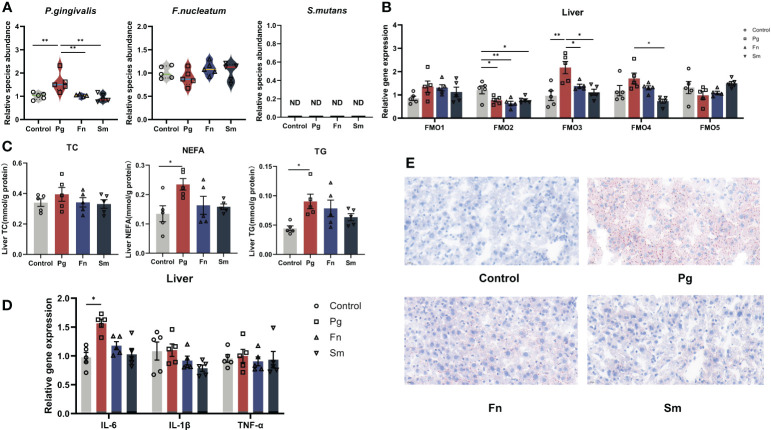
Effect of three oral pathogenic bacteria on the expression of liver flavin-containing monooxygenase 3 (FMO3) enzyme and lipid metabolism. **(A)** Abundance profiles of oral pathogens in mouse liver, quantified through qPCR. **(B)** Expression levels of the FMO gene family involved in TMA oxidase activity in mouse liver, measured by qPCR. **(C)** Expression levels of lipid metabolism factors in mouse liver, assessed using microplate assay kits. **(D)** Expression levels of inflammatory factors in mouse liver, quantified via qPCR. **(E)** Oil Red O staining results depicting lipid accumulation in mouse liver at 400× magnification. Significance levels denoted: *p<0.05, **p<0.01.

TMA originating from the gut, enters to the liver via the bloodstream and undergoes oxidation to TMAO, a process catalyzed by the FMO family, particularly FMO3, in the liver (Shih et al., 2015). To substantiate the effect of oral pathogens on disrupting the gut barrier from the gut to the liver, we also analyzed the abundance of different oral pathogens within the liver, as depicted in **Figure 4A**. After administration of *Pg*, we observed a noteworthy increase in the relative abundance of *Pg* in the *Pg* group compared to the others. However, there were no significant changes in the relative abundance of *Fn* in the livers of the *Fn* group as compared to the control group. Likewise, *Sm* was not detected in the livers of any of the groups (**Figure 4A**).

The pivotal enzyme involved in TMAO production is FMO3, which predominantly resides in the liver. Hence, we assessed the influence of various oral pathogen groups on the expression levels of FMO family members in the liver (**Figure 4B**). FMO3 expression was markedly upregulated in the *Pg* group relative to the control group. Conversely, *Pg*, *Fn*, and *Sm* groups demonstrated a reduction in FMO2 gene expression compared to the control group. FMO4 expression was elevated in the *Pg* group compared to the *Sm* group. Nevertheless, no discernible differences in the relative expression of FMO1 and FMO5 were discerned among the four groups.

To delve deeper into the effects of bacteria on liver fat metabolism, we examined the levels of common molecules linked to lipid metabolism in the liver. Although the AST/ALT liver function ratio was marginally elevated in the in the *Pg* group (**Supplementary Figure 6**), this increase didn’t reach statistical significance when compared across the other groups. Notably, both NEFA and TG levels in the liver were conspicuously higher in the *Pg* group than in the others (**Figure 4C**). Our findings also indicated an upregulation of the liver inflammatory factor IL-6 in the *Pg* group (**Figure 4D**). Additionally, no significant disparities in IL-1β and TNF-α levels were observed among the four groups. To visually discern the precise effects of *Pg* on liver steatosis in mice, we used Oil Red O staining to illustrate the distribution of liver lipid droplets (**Figure 4E**), and the results matched the results mentioned above. It revealed that in the control group, no lipid droplets were observed within hepatocytes, while almost all hepatocytes in the *Pg* group exhibited varying sizes of bright red lipid droplets. In the *Fn* group, the majority of hepatocytes contained lipid droplets of varying sizes. Approximately half of the hepatocytes in the *Sm* group showed a small amount of lipid droplets.

### 3.5 *Pg* upregulates the expression of FMO3 and induces an increase in lipid factors in HepG2 cells

The animal experiment results demonstrated a notable alteration in *Pg* content within the liver among the three oral pathogenic bacteria tested. We used *Pg*-LPS to directly stimulate liver cells, then to further verify the effect of *Pg* on the expression of liver FMO3 and changes in lipid metabolism. *Escherichia coli* was selected as a control in our study due to its prevalence in the gut microbiota and its well-documented involvement in liver disease, providing a relevant baseline for assessing the unique pathogenic effects of *Pg* on liver health.

**Figure 5:**
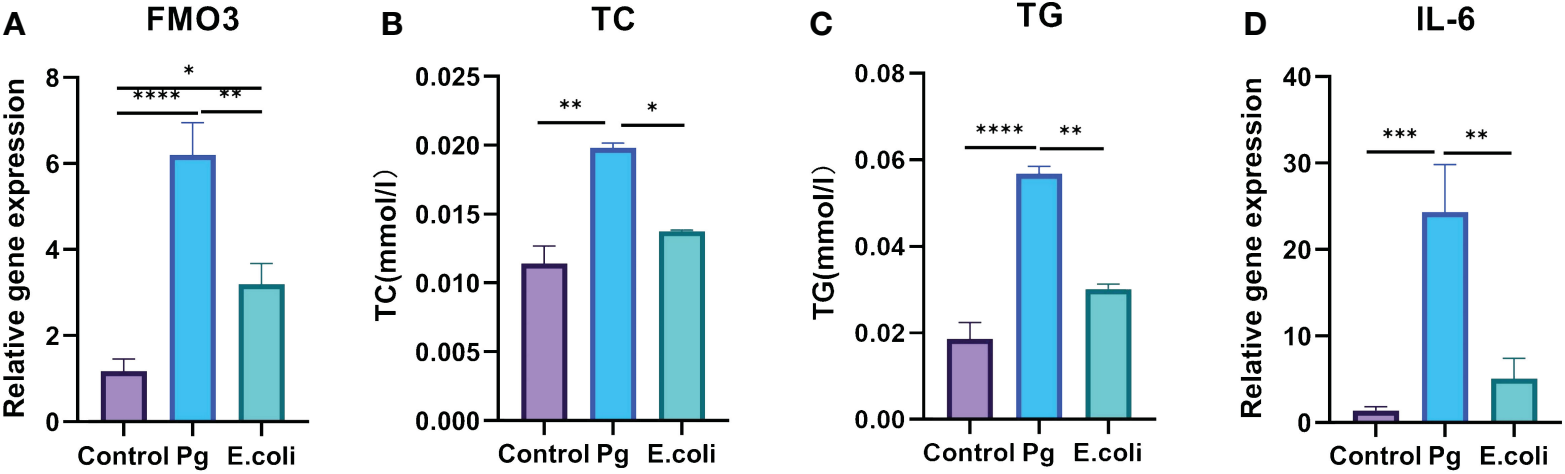
Effect of *Porphyromonas gingivalis* lipopolysaccharide (*Pg*- LPS) on FMO3 enzyme expression and lipid metabolism in HepG2 cells. **(A)** Relative gene expression level of FMO3 in HepG2 cells treated with 10 µg/mL *Pg*-LPS and *Escherichia coli* LPS, determined through qPCR. **(B)** Total cholesterol (TC) levels in HepG2 cells subjected to 10 µg/mL *Pg*-LPS and *E*. *coli-*LPS, assessed using microplate assay kits. **(C)** Triglycerides (TG) levels in HepG2 cells following treatment with 10 µg/mL *Pg*-LPS and *E*. *coli*-LPS, measured with microplate assay kits. **(D)** Relative gene expression level of interleukin-6 (IL-6) in HepG2 cells exposed to 10 µg/mL *Pg*-LPS and *E*. *coli*-LPS, quantified by qPCR. *p<0.05, **p<0.01, ***p<0.001.****p<0.0001.

HepG2 cells were exposed to varying concentrations of *Pg*-LPS and *E. coli*-LPS (0, 10, 50, 100 µg/mL) for 24 hours to compare the virulence effects between *E. coli* and *Pg*. The findings revealed an augmentation in the gene expression levels of FMO3 (**Figure 5A**) and an increase in lipid metabolism markers TC (**Figure 5B**) and TG (**Figure 5C**). Additionally, IL-6 levels were significantly elevated in both LPS-treated groups compared with the control group, particularly in the 10 µg/mL LPS groups (**Figure 5D**). A highly significant difference was also observed between *Pg*-LPS and *E. coli*-LPS groups, indicating distinct responses to these bacterial challenges.

## 4 Discussion

TMAO is a confirmed independent risk factor for NAFLD and CVD, etc (He et al., 2023; Spence, 2023), and it exhibits a bidirectional association with oral diseases (Wang et al., 2022). Our previous research revealed that experimental periodontitis in mice altered the diversity and composition of the gut microbiome, consequently affecting the TMAO levels in plasma (Xiao et al., 2022). So, we further explored the specific changes and possible pathogens associated with TMAO formation process.

An important finding of this study is the significant increase in plasma TMAO concentration in mice following oral administration of *Pg*. This finding is consistent with results reported in previous research on a chronic apical periodontitis animal model induced using *Pg* (Gan et al., 2023). In addition, high levels of betaine and creatinine were observed in the *Pg* group of mice in our study, potentially indicating adaptive responses to liver inflammation (Wang et al., 2021). Interestingly, the other two oral pathogens did not significantly affect TMAO levels, implying a distinct role for *Pg* in promoting TMAO production through the gut-liver axis. This association further indicates the relationship between periodontitis and systematic diseases.

In this study, oral administration of *Pg* in mice caused a significant upregulation of FMO3 gene expression in the liver compared to the other three groups. FMO3 is the vital enzyme in the oxidation of TMA into TMAO, and the *Pg*-induced high TMAO levels can be attributed to the upregulation of FMO3 expression. **Figure 4B** further support that under the background of normal mice, the process of *Pg*-induced elevated plasma TMAO levels depends more on the oxidation of TMA in the liver rather than the generation of TMA in the intestine. *Pg*, *Fn*, and *Sm* groups exhibited a downregulation in FMO2 gene expression compared to the control group, which potentially indicates a general stress response of the liver to oral pathogenic bacterial infection (Ni et al., 2022). *In vitro* TMAO exacerbates lipid accumulation, inflammation, and fibrosis in the liver (Vallianou et al., 2019; Carpino et al., 2020). Moreover, the gut microbiota metabolite TMAO can exacerbate lipid deposition and liver fibrosis in HepG2 cells through the KRT17 *in vitro* (Nian et al., 2023). Elevated TMAO levels can induce oxidative stress, subsequently activating the NLRP3 inflammasome through the PERK-FoxO1-UPR-NF-κB pathway, ultimately inducing liver inflammation (Chen et al., 2019). The findings align with the results observed in our experiments. In our study, mice administered with *Pg* orally exhibited hepatic steatosis, accompanied by increased expression levels of lipid metabolism factors. Additionally, *in vitro* experiments revealed that *Pg* upregulated the expression of FMO3. Similarly, upon *Pg*-LPS stimulation of HepG2 cells, the levels of cellular lipid metabolism factors increased. Previous findings indicate that both TMAO and FMO3 are implicated in dysregulated hepatic lipid metabolism. The genetic ablation of FMO3 (FMO3KO) markedly reduces the levels of TMAO, exhibits lipid-lowering effects, and alleviates thrombosis formation (Shih et al., 2019).

So, we may conclude that the differences in plasma TMAO levels between the *Pg* and other groups, primarily driven by *Pg*’ s enhanced upregulation of hepatic FMO3, which facilitates greater TMA to TMAO conversion. Additionally, *Pg* induces stronger inflammatory responses and alters hepatic lipid metabolism, which could indirectly influence the metabolism of compounds related to TMAO production.

Significant changes in the gut microbiome structure were detected in mice exposed to both *Pg* and *Fn* groups. These changes included a decrease in the proportions of *Firmicutes*/*Bacteroidota*, indicating a potential disruption of gut microbiota balance (Zhang et al., 2022; Ruan et al., 2023). *Muribaculaceae* and *Alloprevotella* genera are related to the intestinal inflammation, barrier dysfunction, as well as glucose and lipid metabolism, further emphasizing the role of these oral pathogens in systemic diseases (Lagkouvardos et al., 2019; Ismael et al., 2022).

However, the variations in gut genera associated with TMAO remain unclear, which is consistent with previous reports, perhaps due to standard diet we used. Apart from that, it is a little bit surprising that the profiles of the microbiota and their associated metabolites—cecal TMA and TMAO—are quite distinct. TMA production in the gut is directly influenced by gut microbiota. Unlike studies that have investigated high-choline or high-fat diets (Agus et al., 2021), where an increased substrate availability might lead to higher and more consistent levels of cecum TMA and TMAO, our mice were on a standard diet. This is relevant when considering the internal consumption of cecum TMA to cecum TMAO. While a major portion of cecum TMAO stems from plasma TMAO, we propose that a segment might also arise from local oxidative processes within the gut, as indicated by the recent literature (Koay et al., 2021), mediated by specific microbiota or intestinal cells under oxidative stress, although further investigation is needed to confirm these pathways. And correlation between plasma TMA and gut microbiota seems to mirror the relationship with cecum TMA due to the translocation process. Moreover, while plasma TMAO correlates with systemic diseases like AS, the associated gut microbes may influence disease progression rather than directly contributing to TMAO production. *Fn* and *Pg* can alter the gut microbiota structure, leading to intestinal inflammation. Although the *Sm* group did not exhibit apparent liver or intestinal symptoms, increased relative abundances of *Alistipes* and *Prevotellaceae_UCG-001* were noted. *Alistipes* is linked to bile acid metabolism and anti-inflammatory properties (Parker et al., 2020), while *Prevotellaceae UCG-001* is positively correlated with AMPK signaling pathway and negatively correlated with markers of glucose and lipid metabolism disorder (Song et al., 2019). These genera are moderately correlated with cecal TMA content; however, their increased presence did not coincide with elevated TMA levels in the cecum or plasma, illustrating complex microbial interactions that may be influenced by other unmeasured factors. These observations can be attributed to the stimulation by the gut microbiota, which may require specific dietary substrates. Furthermore, the production of TMAO in mice on a normal diet may not primarily be mediated by the gut microbiome but rather by changes in the FMO3 enzyme pathway.

Despite being introduced exogenously, *Fn* exhibited only a modest two-fold increase, consistent with its known role in biofilm rather than in a free-floating, planktonic form (Brennan and Garrett, 2019). Surprisingly, both *Pg* and *Fn* were detected in control animals, which can be attributed to *Pg*’s scarce presence in the intestinal tract, primarily entering via oral ingestion (Jia et al., 2024), and *Fn*’s baseline presence as an intestinal resident. Notably, an increased detection of *Pg* in the liver was observed, indicating that *Pg* may partially translocate to the liver (Yao et al., 2023), highlighting the potential systemic effect of oral pathogens. However, this study did not use fluorescent labeling to track pathogenic bacteria, a limitation that future research will address to better understand pathogen dynamics and interactions.

In summary, *Pg* demonstrates a significantly greater effect on increasing peripheral blood TMAO levels compared to *Fn* and *Sm*. This is primarily due to *Pg*’s unique capabilities in disrupting the intestinal barrier and directly stimulating the expression of FMO3 in the liver. Despite minimal differences in intestinal TMA precursors and TMA levels among the groups, *Pg*’s specific biological mechanisms allow it to more effectively influence TMAO production through the gut-liver axis. Our study shows that, compared to the control group, the content of *Pg* in the liver is significantly increased, whereas the changes in *Fn* are not substantial, further supporting that *Pg*’s ability to compromise the intestinal barrier and reach the liver is more pronounced than *Fn*. Additionally, *in vitro* experiments have confirmed that stimulation by *Pg* increases the expression of FMO3 and lipid factors in liver cells. There is also substantial evidence that *Pg* can enter the liver and cause hepatic steatosis (Yoneda et al., 2012; Kuraji et al., 2021; Wang Q. et al., 2022).

In contrast, although *Fn* can also induce intestinal inflammation and changes in the gut microbiota, its impact on TMAO production in healthy individuals under a normal diet is relatively weaker than *Pg*, possibly due to its less disruptive effect on the intestinal barrier. The effect of *Sm* is even less pronounced; significant changes in *Sm* were not detected in the intestines or liver, and while genera related to cecal TMA metabolism appeared to shift in the *Sm* group, the overall diversity of the gut microbiota was more similar to the control group. Existing studies indicate that *Sm* has been demonstrated to enter the systemic circulation and cause pathogenic effects through intravenous injection, yet oral administration does not exacerbate intestinal or hepatic symptoms. aligning with our findings (Kitamoto and Kamada, 2022).

## 5 Conclusion

This study highlights the significant effect of the periodontal pathogen *Pg* on the TMA-TMAO pathway, primarily through modulation of FMO3 expression and regulation of hepatic lipid metabolism, compared to *Fn* and *Sm*. *Pg* uniquely enhances TMAO production, which is closely associated with the progression of systemic diseases, highlighting its potential as a key target in understanding and managing TMAO-related metabolic diseases. Future studies should delve into the molecular mechanisms by which *Pg* regulates FMO3 expression and its effects on lipid metabolism, aiming to uncover new therapeutic avenues for mitigating the effect of high TMAO levels on systemic health.

The similar changes in gut microbiota structure and composition induced by *Pg* and *Fn*, indicate their potential involvement in the influence of periodontitis on intestinal diseases.

## Data availability statement

The datasets generated for this study can be found in the Sequence Read Archive (SRA) SUB14350117. It is associated with BioProject PRJNA1095377.

## Ethics statement

Ethical approval was not required for the studies on humans in accordance with the local legislation and institutional requirements because only commercially available established cell lines were used. The animal study was approved by Nanjing University Animal Experiment Ethics Committee (Approval No. IACUC-D2102033). The study was conducted in accordance with the local legislation and institutional requirements.

## Author contributions

XW: Conceptualization, Data curation, Formal analysis, Funding acquisition, Investigation, Methodology, Project administration, Resources, Software, Supervision, Validation, Visualization, Writing – original draft, Writing – review & editing. LC: Conceptualization, Data curation, Investigation, Methodology, Resources, Supervision, Validation, Writing – review & editing. YT: Conceptualization, Data curation, Methodology, Writing – review & editing. WX: Conceptualization, Resources, Software, Writing – review & editing. LH: Formal analysis, Software, Writing – review & editing. JW: Formal analysis, Supervision, Writing – review & editing. HW: Formal analysis, Funding acquisition, Resources, Writing – review & editing. SX: Conceptualization, Data curation, Formal analysis, Funding acquisition, Investigation, Methodology, Project administration, Resources, Software, Supervision, Validation, Visualization, Writing – review & editing.
