# Supplementary material for: Effect of three oral pathogens on the TMA-TMAO metabolic pathway
Source: Front Cell Infect Microbiol. 2024 May 21;14:1413787. doi: 10.3389/fcimb.2024.1413787 (PMC11148326; doi:10.3389/fcimb.2024.1413787)
Supplement: Supplementary file 1 [file DataSheet_1.pdf]

Supplementary Material

1 Supplementary Data

2 Supplementary Figures and Tables

2.1 Supplementary Figure 1

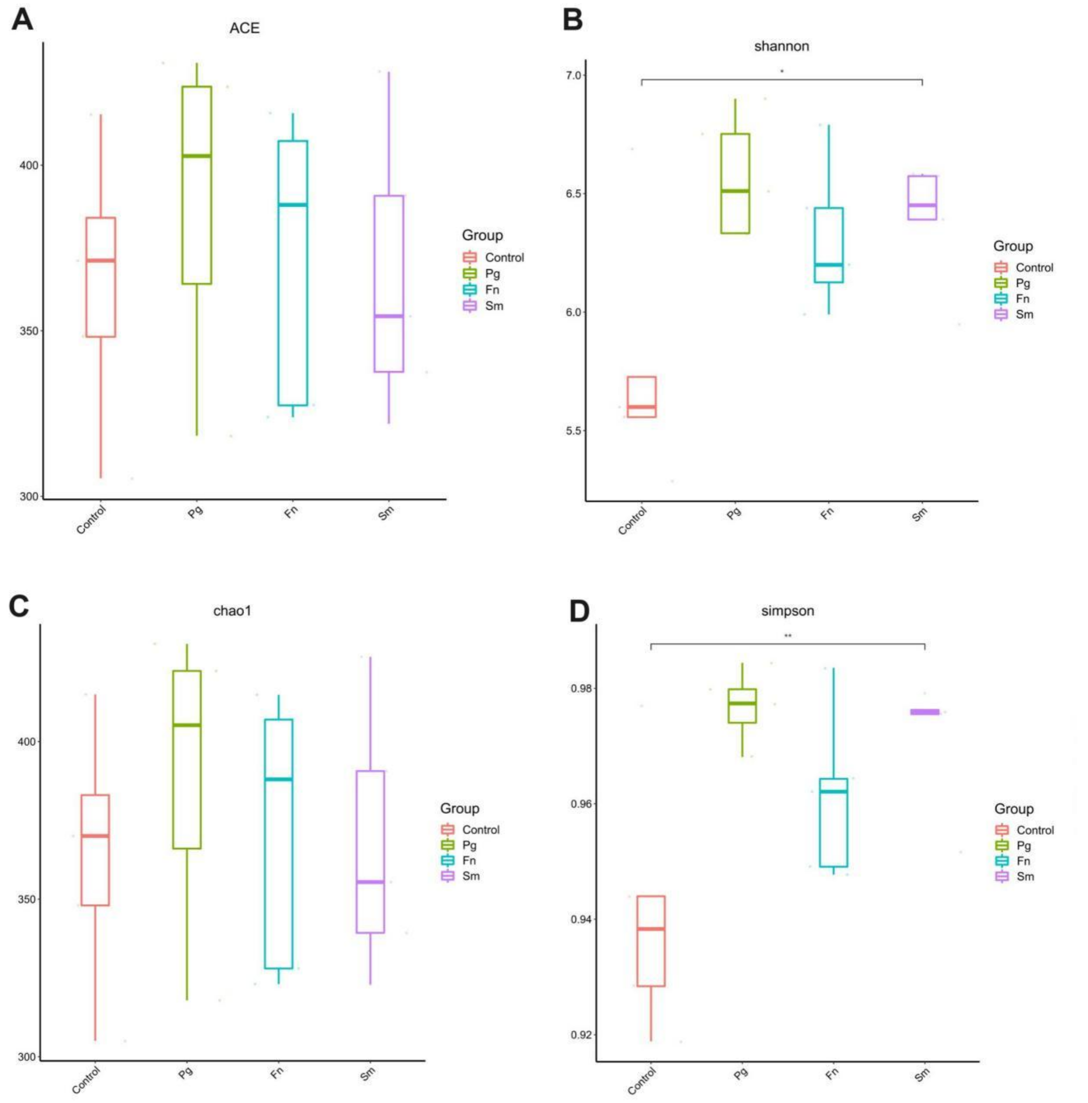

Supplementary Figure 1. Analysis of intestinal flora alpha diversity in different treatment groups. (A) ACE index. **(B)** Shannon index. **(C)** Chao1 index. **(D)** Simpson index.***p<0.001, **p<0.01, *p<0.05

2.2 Supplementary Figure 2

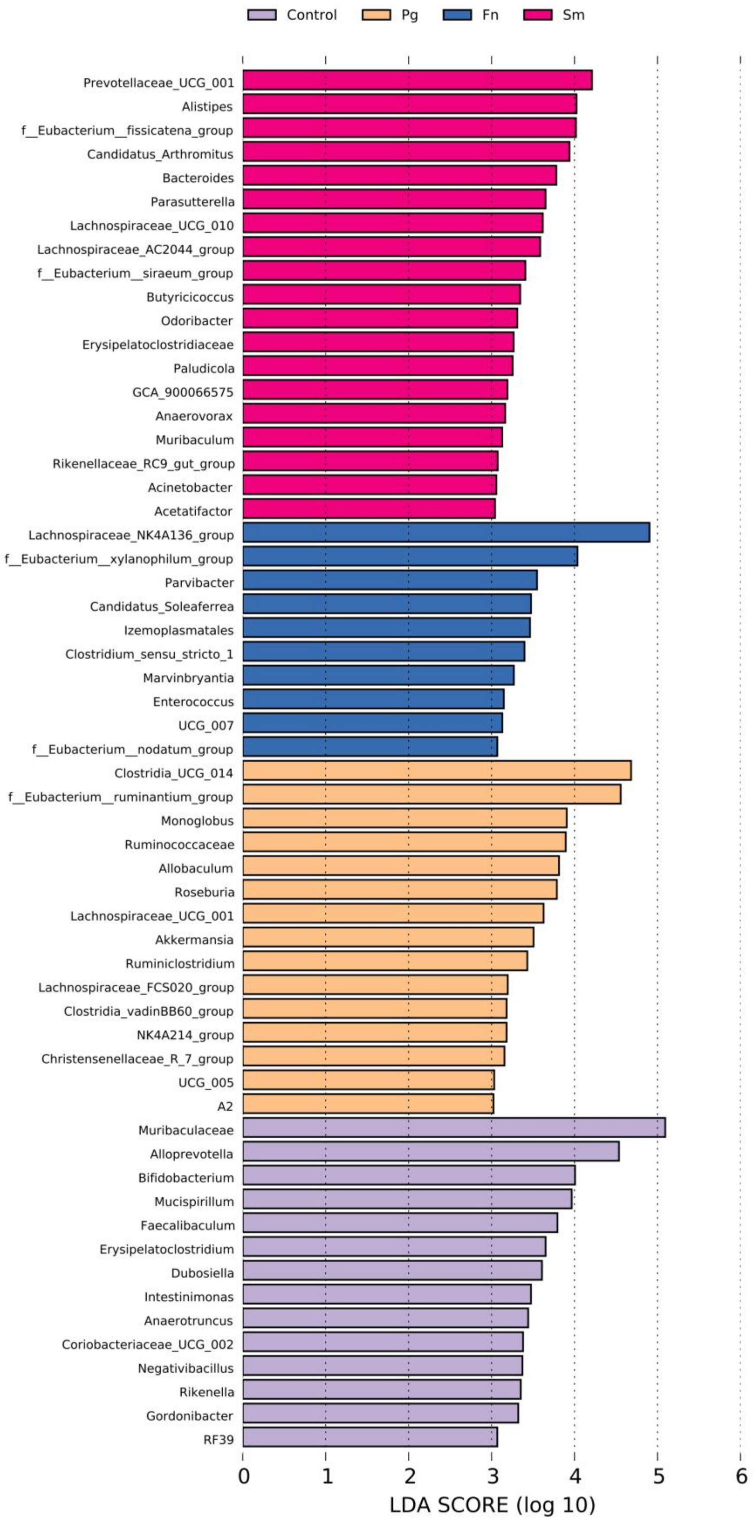

Supplementary Figure 2. LEfSe analysis of different treatment groups.

2.3 Supplementary Figures 3

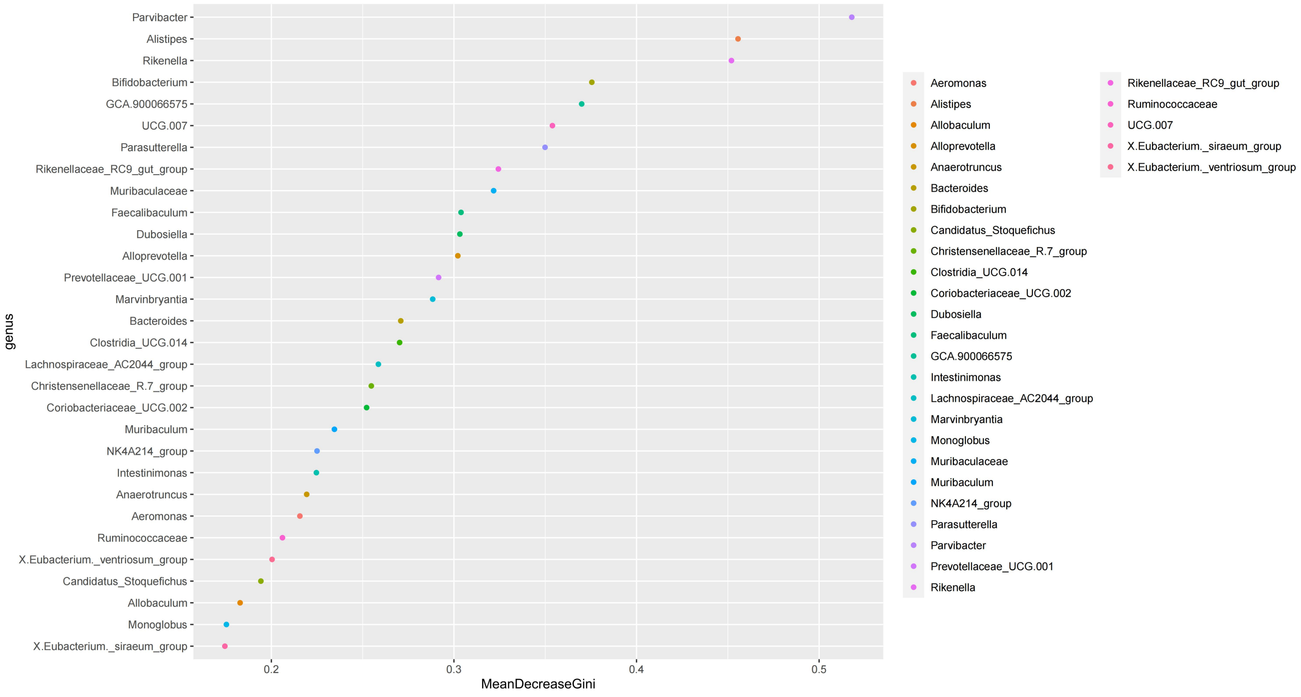

Supplementary Figure 3. Random forest analysis identifies key microbial groups in the gut.

2.4 Supplementary Figure 4

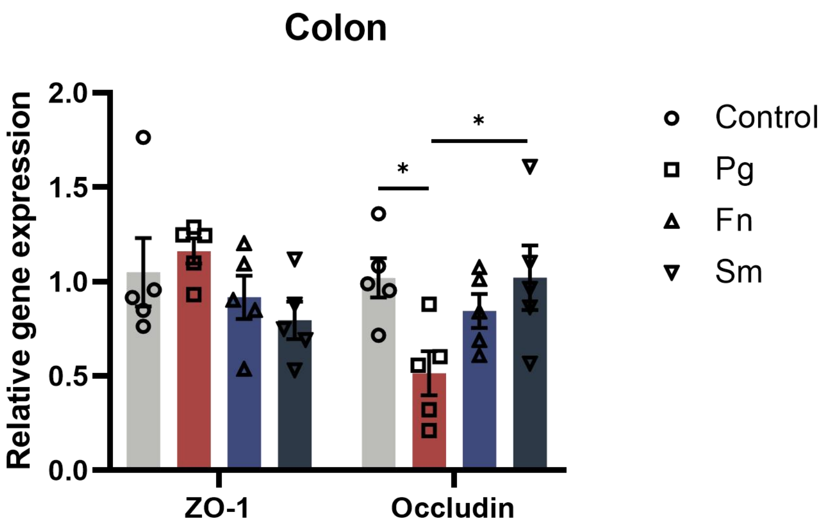

Supplementary Figure 4. Intestinal barrier proteins different treatment groups.

2.5 Supplementary Figure 5

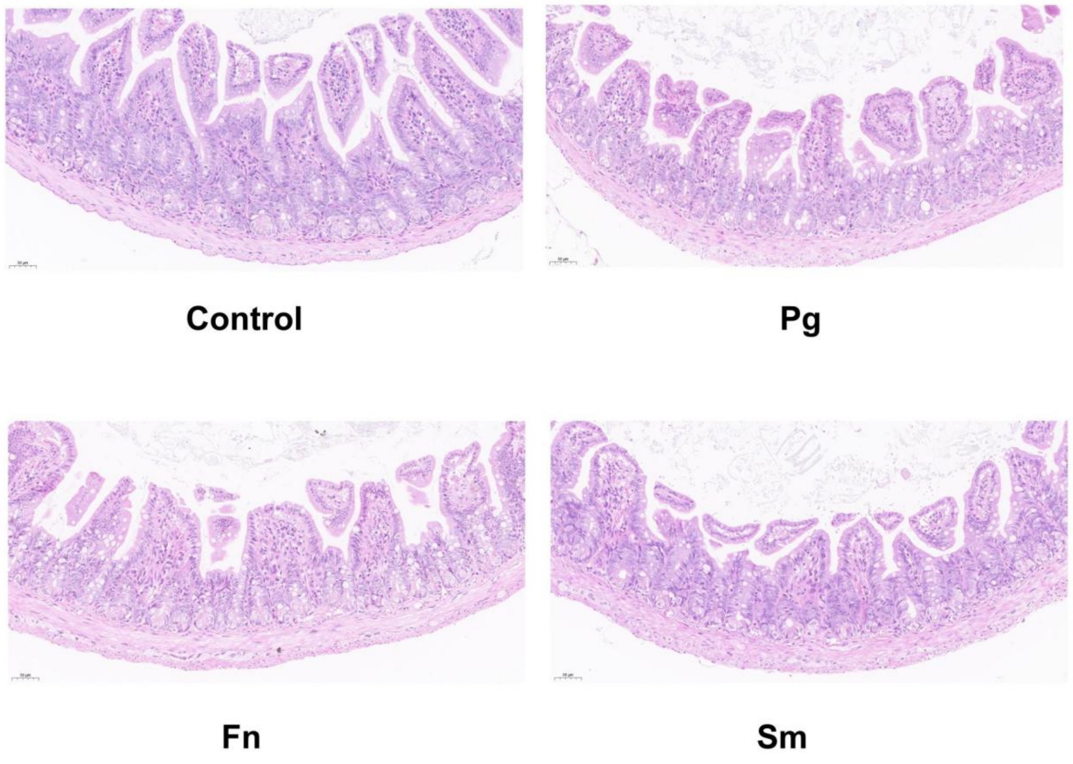

Supplementary Figure 5. Intestinal HE staining (200×) of different treatment groups

2.6 Supplementary Figure 6

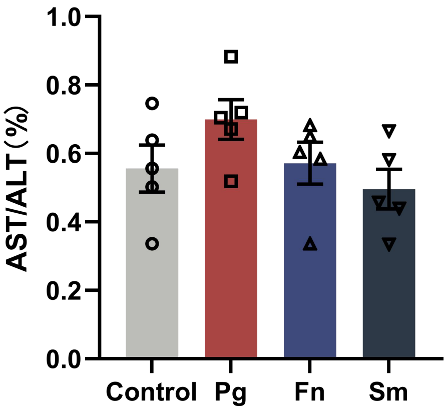

Supplementary Figure 6. Levels of AST/ALT raio in liver of different treatment groups.

2.7 Supplementary Table 1 Primer sequence used in Quantitative Real-time PCR

Supplemental Table. 1. Primer sequence used in Quantitative Real-time PCR

Gene	Forward Primer	Reverse Primer
ZO-1	GCCGCTAAGAGCACAGCAA	TCCCCACTCTGAAAATGAGGA
Occludin	TGAAAGTCCACCTCCTTACAGA	CCGGATAAAAAGAGTACGCTGG
IL-6	TAGTCCTTCCTACCCCAATTTC	TTGGTCCTTAGCCACTCCTT
IL-1β	GCAACTGTTCCTGAACTCAAC	ATCTTTTGGGGTCCGTCAAC
TNF-α	CCCTCACACTCAGATCATCTTC	GCTACGACGTGGGCTACAG
P.gingivalis	AGGCAGCTTGCCATACTGCG	ACTGTTAGCAACTACCGATG
F.nucleatum	CAACCATTACTTTAACTCTACCA	GTTGACTTTACAGAAGGAGATTA TGT
S.mutans	CCTCAGGAAAGTCTGGAGTAAA AGCCTA	GCCTTAGCTCCCCCACTAACCC
Universal primers	AGAGTTTGATCCTGGCTCAG	TACGGCTACCTTGTTACGACTT
FMO1	ACAGCCGACAGTATAAACATC	CCCTCCAGTAGTGCTGAGGAA
FMO2	AGTGGCCTAATCTCTCTGAAG	CATCGGGAAGTCACTGAAACA
FMO3	ACTGGTGGTACACAAGGCAG	ATGGTCCCATCCTCAAACACA
FMO4	GATTGGAGCTGGCGTAAGTG	TGTCAGCAAACTTCCACAGTC
FMO5	GAGGGCTTGGAACCTGTCTG	CACGGACTGGTAAATACTGGC
ACTB	GGCTGTATTCCCCTCCATCG	CCAGTTGGTAACAATGCCATG
Human-FMO3	CATCATGTGTATCCCAACCTACC	CTGGTTCTTTATAGTCCCTGCTG
Human-IL-6	ACTCACCTCTTCAGAACGAATTG	CCATCTTTGGAAGGTTCAGGTTG
Human-ACTB	GGAGCGAGATCCCTCCAAAAT	GGCTGTTGTCATACTTCTCATGG
